# Letter from the Editor in Chief

**DOI:** 10.19102/icrm.2020.110302

**Published:** 2020-03-15

**Authors:** Moussa Mansour


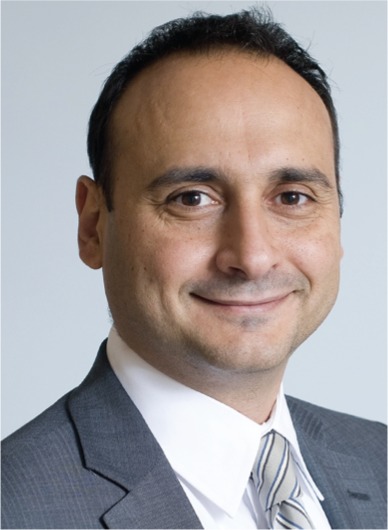


Dear Readers,

The application of ionizing radiation during interventional cardiac procedures is detrimental to both patients and operators alike. A report in 2006 found that radiation exposure among patients had more than doubled since the 1980s,^1^ with even higher levels likely reached since then. In the literature, the evidence supports a clear association between radiation exposure and cancer.^2^ Among patients receiving a 100-mSv effective dose, approximately one in 200 will exhibit a fatal cancer and one in 100 will experience a cancer that may or may not be fatal.^3^ In addition to the potential impact of radiation on the patient, there is a significant risk of adverse effects faced by the operator, with research reporting an association between professional radiation exposure and the risk of developing left-sided brain cancer.^4^

In the past five years, the trend of using zero or near-zero fluoroscopy during cardiac procedures has gained traction. In particular, advances in technology such as intracardiac echocardiography, advanced three-dimensional electroanatomical systems, and novel transseptal access tools have supported the growth of this trend. This issue of *The Journal of Innovations in Cardiac Rhythm Management* contains a comprehensive review of the state of fluoroless procedures in cardiac electrophysiology practice.^5^ The authors summarize the key available studies investigating the adoption of zero or near-zero fluoroscopy and, more importantly, provide detailed step-by-step descriptions of how to perform complex procedures without the use of radiation. I hope that you enjoy reading the article and the remaining content of this issue.

I also would like to take this opportunity to thank the editorial team, section editors, and reviewers of *The Journal of Innovations in Cardiac Rhythm Management* for the roles they played leading up to the recent achievement of PubMed indexing for the journal. This is an important step in the journey of this young journal and would not have been possible without their hard work, dedication, and expertise. Thank you as well to the readers for your continued support.

Sincerely,


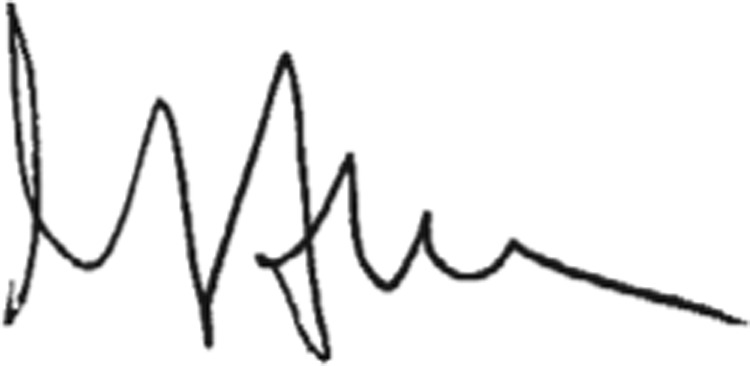


Moussa Mansour, md, fhrs, facc

Editor in Chief

The Journal of Innovations in Cardiac Rhythm Management

MMansour@InnovationsInCRM.com

Director, Atrial Fibrillation Program

Jeremy Ruskin and Dan Starks Endowed Chair in Cardiology

Massachusetts General Hospital

Boston, MA 02114
